# Early epidemiological investigations: World Health Organization UNITY protocols provide a standardized and timely international investigation framework during the COVID‐19 pandemic

**DOI:** 10.1111/irv.12915

**Published:** 2021-10-05

**Authors:** Isabel Bergeri, Hannah C. Lewis, Lorenzo Subissi, Anthony Nardone, Marta Valenciano, Brianna Cheng, Ketevan Glonti, Bridget Williams, Ibukun‐Oluwa Omolade Abejirinde, Alice Simniceanu, Alessandro Cassini, Rebecca Grant, Angel Rodriguez, Andrea Vicari, Lubna Al Ariqi, Tasnim Azim, Pushpa Ranjan Wijesinghe, Soatiana Cathycia Rajatonirina, Joseph Chukwudi Okeibunor, Linh‐Vi Le, Mark Katz, Aisling Vaughan, Pernille Jorgensen, Gudrun Freidl, Richard Pebody, Maria D. Van Kerkhove

**Affiliations:** ^1^ WHO Health Emergencies Programme World Health Organization Headquarters Geneva Switzerland; ^2^ Epidemiology Departement Epiconcept Paris France; ^3^ Pan American Health Organization Washington D.C. USA; ^4^ World Health Organization for the Eastern Mediterranean Cairo Egypt; ^5^ Regional Office for South‐East Asia World Health Organization New Delhi India; ^6^ Regional Office for Africa World Health Organization Brazzaville Republic of the Congo; ^7^ Regional Office for the Western Pacific World Health Organization Manila Philippines; ^8^ Regional Office for Europe World Health Organization Copenhagen Denmark

**Keywords:** COVID‐19, infectious diseases, SARS‐CoV‐2, seroepidemiology, serology, seroprevalence

## Abstract

**Background:**

The declaration of Coronavirus disease 2019 (COVID‐19) as a Public Health Emergency of International Concern (PHEIC) on 30 January 2020 required rapid implementation of early investigations to inform appropriate national and global public health actions.

**Methods:**

The suite of existing pandemic preparedness generic epidemiological early investigation protocols was rapidly adapted for COVID‐19, branded the ‘UNITY studies’ and promoted globally for the implementation of standardized and quality studies. Ten protocols were developed investigating household (HH) transmission, the first few cases (FFX), population seroprevalence (SEROPREV), health facilities transmission (n = 2), vaccine effectiveness (n = 2), pregnancy outcomes and transmission, school transmission, and surface contamination. Implementation was supported by WHO and its partners globally, with emphasis to support building surveillance and research capacities in low‐ and middle‐income countries (LMIC).

**Results:**

WHO generic protocols were rapidly developed and published on the WHO website, 5/10 protocols within the first 3 months of the response. As of 30 June 2021, 172 investigations were implemented by 97 countries, of which 62 (64%) were LMIC. The majority of countries implemented population seroprevalence (71 countries) and first few cases/household transmission (37 countries) studies.

**Conclusion:**

The widespread adoption of UNITY protocols across all WHO regions indicates that they addressed subnational and national needs to support local public health decision‐making to prevent and control the pandemic.

## 1 BACKGROUND

In December 2019, a novel coronavirus, severe acute respiratory syndrome coronavirus 2 (SARS‐CoV‐2), responsible for coronavirus disease 2019 (COVID‐19), was first identified in Wuhan, China, from where it rapidly spread worldwide COVID‐19 was declared as a public health emergency of international concern (PHEIC) by the World Health Organization (WHO) on 30 January 2020.

One of the key difficulties for decision makers in the early phase of any epidemic of a novel pathogen is to determine the appropriate public health measures in the absence of information on transmission characteristics, extent of infection, severity, and scale of the threat. Following a review of the global response to the last pandemic (2009 influenza pandemic H1N1),^1^ the global Consortium for the Standardization of Influenza Seroepidemiology (CONSISE)^2^ and WHO's Influenza Pandemic Special Investigations and Studies (IPSS) initiative were established to develop a suite of standardized early investigation protocols,^3^ supported by the Global Influenza Programme^4^ and the Pandemic Influenza Preparedness (PIP) Framework.^5^ Standardized protocols were also implemented following the emergence of Middle East Respiratory Syndrome coronavirus (MERS‐CoV) in 2012^6^ and Zika virus in 2016^7^ with delineation of a comprehensive global research agenda.^8^

Building on these global pandemic preparedness efforts, we describe the rapid adaptation and implementation of WHO early investigation protocols for COVID‐19, branded the ‘UNITY studies’,^9^ to generate local data to better inform appropriate national and global public health actions and guidelines. The on‐going and future value of such studies for pandemic preparedness and response is discussed.

## 2 METHODS

### 2.1 Overall concept

UNITY studies enable all countries to rapidly and systematically collect standardized robust data on key epidemiological, virological, and clinical parameters to understand key characteristics of SARS‐CoV‐2.

### 2.2 Embedment into global WHO COVID strategic plan, financing, and accountability

The UNITY initiative is embedded into both 2020 and 2021 WHO's COVID‐19 Strategic Preparedness Response Plans (SPRP) and COVID‐19 Monitoring and Evaluation framework.^10^ Financial support from May 2021 (see acknowledgements) was instrumental for LMIC.

### 2.3 Generic protocol development

Ten generic protocols using eight study methodologies were developed (three adapted from available IPSS protocols). These investigate household (HH) transmission, the first few cases (FFX) and their contacts, population seroprevalence (SEROPREV), health facility transmission (two protocols), vaccine effectiveness (two protocols), pregnancy outcomes and transmission, school transmission, and surface contamination. More information on each protocol is described in Table 1.

**TABLE 1:** UNITY protocols description by publication date on the World Health Organization (WHO)'s website and number of countries intending to or having implemented UNITY protocols^9^ by investigation type, as of 30 June 2021

Protocols name	Description	Date of first publication of protocol on WHO website	Number of countries implementing study			Number of countries intending to implement study
			Total	LMIC (% of total)	HIC (% of total)	
First Few X (FFX) cases and contacts transmission	A prospective case‐ascertained investigation of all identified close contacts of laboratory‐confirmed infections to characterize the transmission dynamics and clinical spectrum of COVID‐19 infection in the general population	31/01/2020	24	17 (71%)	7 (29%)	3
Household (HH) transmission	A prospective investigation of household contacts of laboratory‐confirmed COVID‐19 cases to reinforce the estimates of transmission dynamics and clinical spectrum of infection obtained from the FFX studies for the general population.	25/01/2020	13	5 (38%)	8 (62%)	1
Health facility transmission	Two protocols available to assess the transmissibility of and risk factors for COVID‐19 infection among health workers:		36	25 (69%)	11 (31%)	17
	Case–control study	01/02/2020^a^				
	Prospective cohort study	26/05/2020				
Surface contamination and transmission	A practical “how to” guide on surface sampling for COVID‐19 virus to estimate the persistence of contamination on different surfaces and their possible role in onward transmission.	20/02/2020	‐ ‐	‐ ‐	‐	‐
Population‐based age‐stratified seroprevalence (SEROPREV)	Both cross‐sectional and longitudinal cohort designs to conduct serological surveys to estimate cumulative incidence in the population by age and sex.	19/03/2020	71	50 (70%)	21 (30%)	27
School transmission	A prospective case‐ascertained investigation of school contacts of a laboratory‐confirmed case of COVID‐19 to understand the dynamics of infection among students and staff of schools and childcare institutions	30/11/2020	‐	‐	‐	‐
Maternal, pregnancy and neonatal outcomes and transmission	A prospective cohort study to determine if SARS‐CoV‐2 infection during pregnancy increases the risk of adverse pregnancy, post‐partum or neonatal outcomes^a^	04/12/2020^a^	10	8 (80%)	2 (20%)	9
Vaccine effectiveness	Two protocols available to measure product‐specific COVID‐19 vaccine effectiveness (VE):		17	2 (13%)	15 (87%)	1
	Test negative case–control in hospitalized severe acute respiratory infections (SARI) patients	18/03/2021				
	Prospective cohort study among health workers	04/05/2021				

Abbreviations: HIC, high‐income country; LMIC, low‐ and middle‐income country.

^a^ Multi‐centre studies.

Four protocols were designed from inception to be multi‐country and multi‐centre research studies with sharing of anonymised individual level data, a subsequent planned pooled analysis and related tools including joint data platforms (see Table 1). Remaining protocols were mostly considered to be enhanced surveillance protocols by implementing countries.

### 2.4 Implementation strategy

**FIGURE 1:**
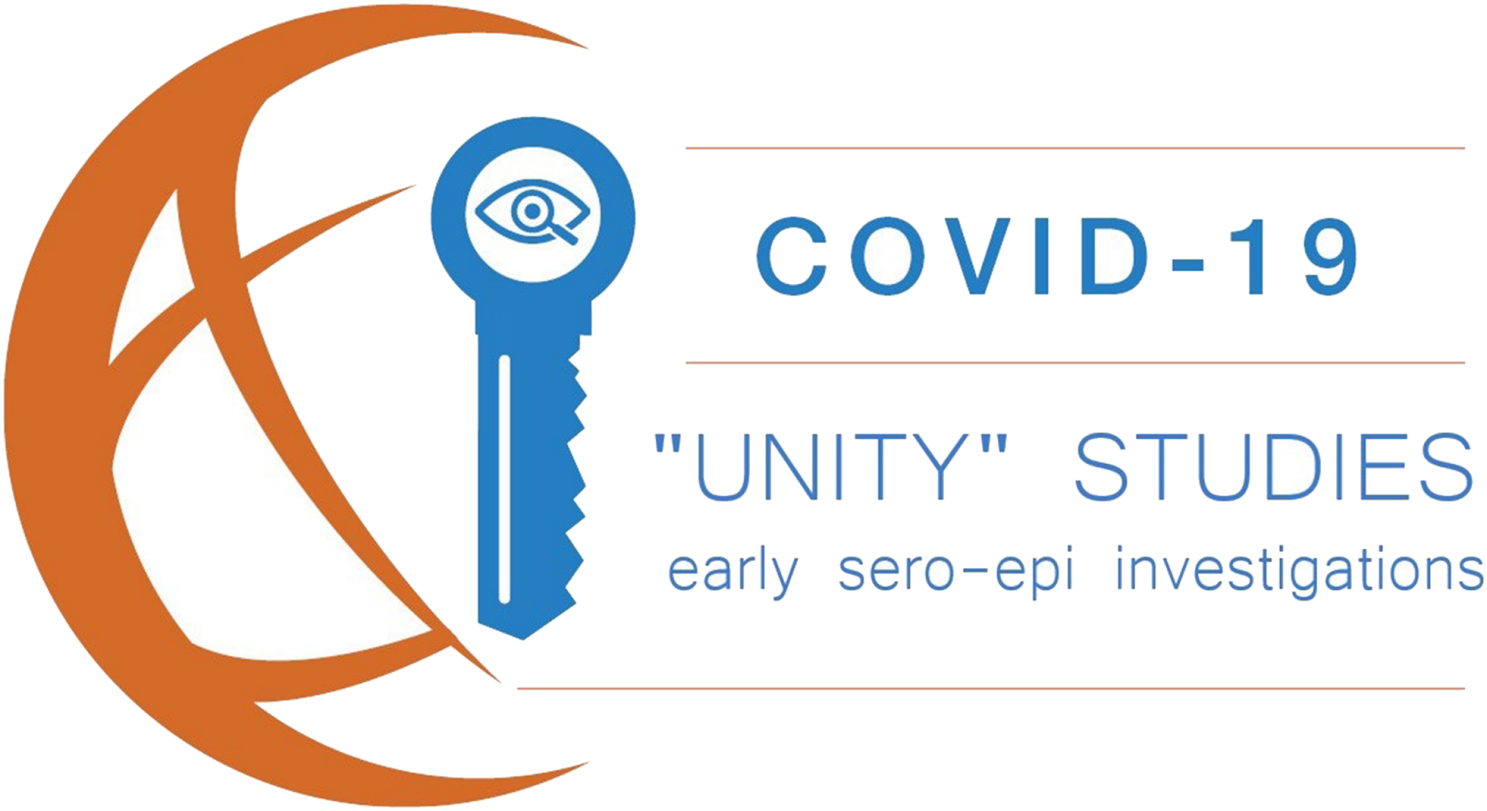
Insignia of WHO UNITY early investigations initiative

WHO Headquarter (HQ), Regional Offices (RO) and Country Offices (CO) and its Special research programmes, supported the dissemination and implementation of protocols to countries, with translation in the six official languages of WHO. This was in interaction with Ministries of Health, local institutions, and through existing collaborations with relevant disease networks and national and international partners (e.g., academic institutions, US‐CDC, and Institut Pasteur). Dissemination was also through United Nations and WHO information products and publications including the WHO website and a generic WHO email address. An insignia (Figure 1) was designed to foster belonging to a join global effort.

### 2.5 Comparability of results through standardization and direct country support

WHO offered substantial technical, financial, and material support to national investigation teams to implement quality UNITY studies, particularly in LMIC. Technical support included scientific review of locally adapted protocols, provision of tools including the option of using Go.Data software^11^), and guidance for field implementation and quality monitoring, advice on laboratory methods, and assistance with data analysis. Training in scientific writing was provided to national teams to facilitate public dissemination of results.

To enhance the comparability of different studies, WHO also advocated for the standardization of serology laboratory methods. Serology testing was standardized through various means: from March 2021 central WHO procurement of manual total antibody ELISAs targeting the receptor‐binding domain (RCB) of SARS‐CoV‐2 spike (S) protein and that demonstrated sensitivity ≥95% and specificity ≥99% in multiple independent evaluations including also samples from mild and asymptomatic infections^12^; provision of research reagent and panel from April 2020^13^; comparative performance evaluation of commercially available assays from Dec 2020^14^; and availability of International Standard by December 2020.^13^

As collaborators undertaking UNITY investigations, national teams were asked to share aggregate results in standardized templates while being assured that ownership of primary data rested firmly with the implementing countries and institutions.

### 2.6 Monitoring of country adoption of protocols

WHO ROs and COs provided weekly updates on the number of countries adopting UNITY studies, defined as either ‘intending to undertake’ or ‘having implemented’ a protocol. ‘Implementation’ was defined as a nationally adapted protocol scientifically validated by WHO, national ethical approval obtained (or waived), and initiation of the investigation (i.e., at least one participant enrolled or one sample taken).

## 3 RESULTS

**FIGURE 2:**
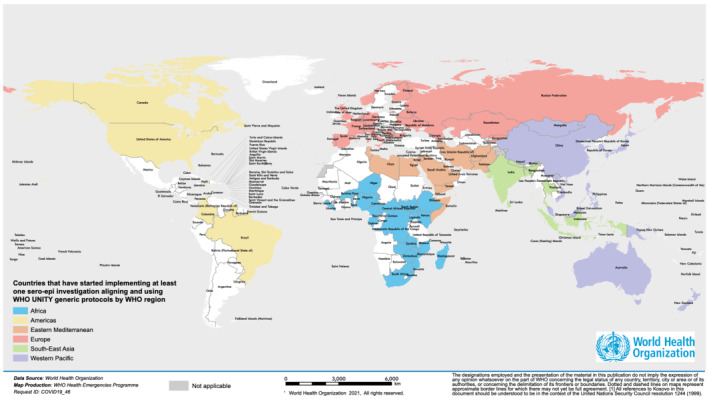
Countries that have started implementing at least one sero‐epidemiological investigation aligned with WHO UNITY generic protocols, from Jan 2020 to 30 June 2021

Five of the ten protocols were published on the WHO website within the first 3 months of response (before 1 April 2020). As of 30 June 2021, 129 countries registered intention to undertake investigations aligned with WHO UNITY studies, and 172 studies had been implemented by 97 countries of which 64% (62/97) were low‐ and middle‐income countries (LMIC) (Table 1 and Figure 2). Studies have been implemented in all WHO regions and in half (53%, 34/64) of all Humanitarian Response Plan (HRP) countries.^15^

The most commonly implemented investigation was the SEROPREV investigation, which has been implemented by 71 countries (Table 1), followed by FFX and HH transmission investigations (n = 37), this last one particularly in the early stage of the pandemic. Health facility transmission investigations were implemented in 36 countries. The monitoring of countries which aligned with or adopted the school or surface contamination protocol was not possible with our human resource capacity at that time. Nevertheless, we retrospectively assess that their uptake was likely low for various reasons including the pandemic dynamic. The pregnancy outcomes and transmission protocol and the two vaccine effectiveness protocols were developed and published at later stage, in December 2020 and March 2021, respectively, to respond to emergent scientific priorities, including to measure the performance of newly implemented vaccine programmes. As of 30 June 2021, 39 countries across all six WHO Geographic Regions (most being LMIC) have been supplied by a centrally procured manual ELISA tests to promote standardization of results.

National teams are sharing standardized results in the templates provided. UNITY studies has enabled countries to more accurately estimate epidemiological indicators, such as transmission and severity parameters, risk factors, seroprevalence, cumulative incidence and secondary attack and infection rates, thereby enhancing routine surveillance.

## 4 DISCUSSION

The widespread adoption of UNITY protocols across all WHO regions indicates that they have addressed subnational and national needs to collect critical data to support local and regional public health decision‐making to prevent and control the pandemic. The quarterly monitoring of the implementation of UNITY investigations as a performance indicator of the COVID‐19 SPRP^10^ further reinforces their significance and impact to also inform global normative work.

WHO rapidly adapted and established the suite of protocols as part of its pandemic preparedness and response planning, which were rapidly adopted by countries. FFX and HH investigations implementation was more relevant for country action early in the pandemic to robustly define key epidemiological and severity parameters of the infection. Nonetheless, the continued collection of such data can provide important insights into the transmission of variants viruses of interest or of concern. It is likely that SEROPREV investigations were of higher priority for countries for rapid policy decision making (and particularly in countries with low testing capacities or weak case‐based reporting system) than for other less used protocols (e.g., school transmission and surface contamination). The assessment of uptake of the more recently developed pregnancy and Vaccine Effectiveness protocols will require more time.

The large number of LMIC, including those affected by armed conflict and political instability (HRP countries), that adopted a UNITY protocol is of note and indicates the value of having readily available, generic protocols that can be adapted for various settings. The number of adopted UNITY studies in this analysis is likely to be under‐reported as institutions may have employed the publicly available protocols independent of WHO support or knowledge, particularly in HIC.

The first set of significant funding support arrived 4 months after the first protocols publication which hampered timely implementation in LMIC. Tight deadlines for fund expenditure from effective reception (ex: less than 3.5 months) was an issue for such activities.

The provision of technical, financial, and laboratory assay support by WHO and international partners and using existing surveillance and research networks was instrumental in the implementation of timely, quality, and standardized studies to better understand COVID‐19 transmission and extent of infection. The support provided enabled countries, regardless of economic status, to build on routine surveillance and develop research capacities, to further scientific learning and support evidence‐based response decisions at local, national, regional and international level. Laboratory standardization has been key to enable inter‐country comparisons. Main lessons learned are described in Box 1.

**Box 1. Lessons learned from the WHO UNITY framework during the COVID‐19 pandemic**

Lessons learned:

- Need for a standardized global framework for epidemiological and laboratory investigations, with ready‐to‐be used, easily adaptable tools, which are adaptable to any resource setting and which can allow inter‐country comparisons
- Operationnal suite of tools supported country enhanced surveillance, the strengthening research capacities independently to resource setting, and in addressing national, regional and global knowledge gaps
- Such framework could be valuable and replicated for any emerging and re‐emerging pathogen pandemic readiness and preparedness plans
- Timely and pooled fund availability to ensure equitable support is instrumental in LMIC
- Building on existing networks of partners, research groups, surveillance systems and public health services were essential for the feasibility of such initiatives

WHO is working with UNITY partners to summarise available study results and to conduct pooled analyses where relevant, by studying populations and at regional and global levels.

An after action review and evaluation of the UNITY initiatives and its protocols to assess their utility to countries in responding to this pandemic and whether they fulfilled key initial objectives started in September 2021 and will be valuable to inform future pandemic preparedness, readiness and response strategy to any respiratory pathogens.

## AUTHOR CONTRIBUTIONS

**Isabel Bergeri:** Conceptualization; formal analysis; funding acquisition; investigation; methodology; project administration; resources; supervision; visualization. **Hannah C. Lewis:** Conceptualization; formal analysis; investigation; methodology; project administration; supervision. **Lorenzo Subissi:** Conceptualization; formal analysis; investigation; methodology; project administration; resources; supervision. **Anthony Nardone:** Conceptualization; formal analysis; investigation; methodology; supervision. **Marta Valenciano:** Conceptualization; formal analysis; investigation; methodology; supervision. **Brianna Cheng:** Investigation; project administration; resources; visualization. **Ketevan Glonti:** Investigation; project administration; resources; visualization. **Bridget Williams:** Conceptualization; investigation; project administration; resources; visualization. **Ibukun‐Oluwa Omolade Abejirinde:** Formal analysis; funding acquisition; investigation; methodology; project administration; resources; supervision. **Alice Simniceanu:** Formal analysis; funding acquisition; investigation; methodology; project administration; resources; supervision. **Alessandro Cassini:** Formal analysis; methodology; project administration; resources; supervision. **Rebecca Grant:** Conceptualization; funding acquisition; methodology. **Angel Rodriguez:** Formal analysis; investigation; project administration; supervision. **Andrea Vicari:** Conceptualization; funding acquisition; investigation; methodology; project administration; resources; supervision. **Lubna Al Ariqi:** Conceptualization; formal analysis; funding acquisition; investigation; methodology; project administration; resources; supervision. **Tasnim Azim:** Conceptualization; funding acquisition; investigation; project administration; resources; supervision. **Pushpa Ranjan Wijesinghe:** Funding acquisition; investigation; project administration; supervision. **Soatiana Cathycia Rajatonirina:** Conceptualization; formal analysis; funding acquisition; investigation; project administration; resources; supervision. **Joseph Chukwudi Okeibunor:** Funding acquisition; investigation; project administration; resources; supervision. **Linh‐Vi Le:** Formal analysis; funding acquisition; investigation; project administration; resources; supervision. **Mark Katz:** Formal analysis; investigation; methodology; project administration; supervision. **Aisling Vaughan:** Formal analysis; investigation; project administration; resources; supervision. **Pernille Jorgensen:** Formal analysis; investigation; project administration; supervision. **Gudrun Freidl:** Formal analysis; investigation; project administration; resources; supervision. **Richard Pebody:** Conceptualization; formal analysis; funding acquisition; investigation; methodology; project administration; supervision. **Maria D van Kerkhove:** Conceptualization; funding acquisition; methodology; project administration; supervision.

5

### PEER REVIEW

The peer review history for this article is available at https://publons.com/publon/10.1111/irv.12915.
